# Experimental feasibility of spectral photon-counting computed tomography with two contrast agents for the detection of endoleaks following endovascular aortic repair

**DOI:** 10.1007/s00330-017-5252-7

**Published:** 2018-02-19

**Authors:** Julia Dangelmaier, Daniel Bar-Ness, Heiner Daerr, Daniela Muenzel, Salim Si-Mohamed, Sebastian Ehn, Alexander A. Fingerle, Melanie A. Kimm, Felix K. Kopp, Loic Boussel, Ewald Roessl, Franz Pfeiffer, Ernst J. Rummeny, Roland Proksa, Philippe Douek, Peter B. Noël

**Affiliations:** 1 Department of Diagnostic and Interventional Radiology, Technische Universität München, Ismaningerstr. 22, 81675 Munich, Germany; 2 CREATIS, CNRS UMR 5220, INSERM U1206, INSA, Lyon, France; 3 Philips GmbH Innovative Technologies, Research Laboratories, Hamburg, Germany; 4 Radiology Department, Lyon University Hospital, Lyon, France; 5 University Lyon1 Claude Bernard, Lyon, France; 6 Chair of Biomedical Physics, Department of Physics and Munich School of BioEngineering, Technische Universität München, 85748 Garching, Germany

**Keywords:** Computed tomography, Photon counting, Endoleak, Iodine, Gadolinium

## Abstract

**Objectives:**

After endovascular aortic repair (EVAR), discrimination of endoleaks and intra-aneurysmatic calcifications within the aneurysm often requires multiphase computed tomography (CT). Spectral photon-counting CT (SPCCT) in combination with a two-contrast agent injection protocol may provide reliable detection of endoleaks with a single CT acquisition.

**Methods:**

To evaluate the feasibility of SPCCT, the stent-lined compartment of an abdominal aortic aneurysm phantom was filled with a mixture of iodine and gadolinium mimicking enhanced blood. To represent endoleaks of different flow rates, the adjacent compartments contained either one of the contrast agents or calcium chloride to mimic intra-aneurysmatic calcifications. After data acquisition with a SPCCT prototype scanner with multi-energy bins, material decomposition was performed to generate iodine, gadolinium and calcium maps.

**Results:**

In a conventional CT slice, Hounsfield units (HU) of the compartments were similar ranging from 147 to 168 HU. Material-specific maps differentiate the distributions within the compartments filled with iodine, gadolinium or calcium.

**Conclusion:**

SPCCT may replace multiphase CT to detect endoleaks without sacrificing diagnostic accuracy. It is a unique feature of our method to capture endoleak dynamics and allow reliable distinction from intra-aneurysmatic calcifications in a single scan, thereby enabling a significant reduction of radiation exposure.

**Key Points:**

• *SPCCT might enable advanced endoleak detection.*

• *Material maps derived from SPCCT can differentiate iodine, gadolinium and calcium.*

• *SPCCT may potentially reduce radiation burden for EVAR patients under post-interventional surveillance.*

## Introduction

An abdominal aortic diameter of 3 cm or more is considered as an abdominal aortic aneurysm (AAA). AAAs have a prevalence of 1.6–7.2% [1, 2] and an incidence of 0.4–0.7% per year in the Western population [3]. Symptomatic aneurysms (e.g. abdominal pain) and rapid growing aneurysms (more than 5 mm in 6 months) [4] as well as AAAs measuring greater than 5.5 cm at baseline are in need to be treated [5]. Next to open surgery, minimal invasive implantation of a covered, self-expandable stent graft—the endovascular aortic repair (EVAR)—is well established since it was first presented by Parodi et al. in 1991 [6]. An endoleak is the most frequent complication (53% of all complications; incidence of 11.7%) following EVAR [7] and typically requires a secondary intervention [8], since it promotes further growing of the aneurysm (in 41% of patients), which can disastrously lead to aortic rupture (in 2.4%) [7].

**Fig. 1:**
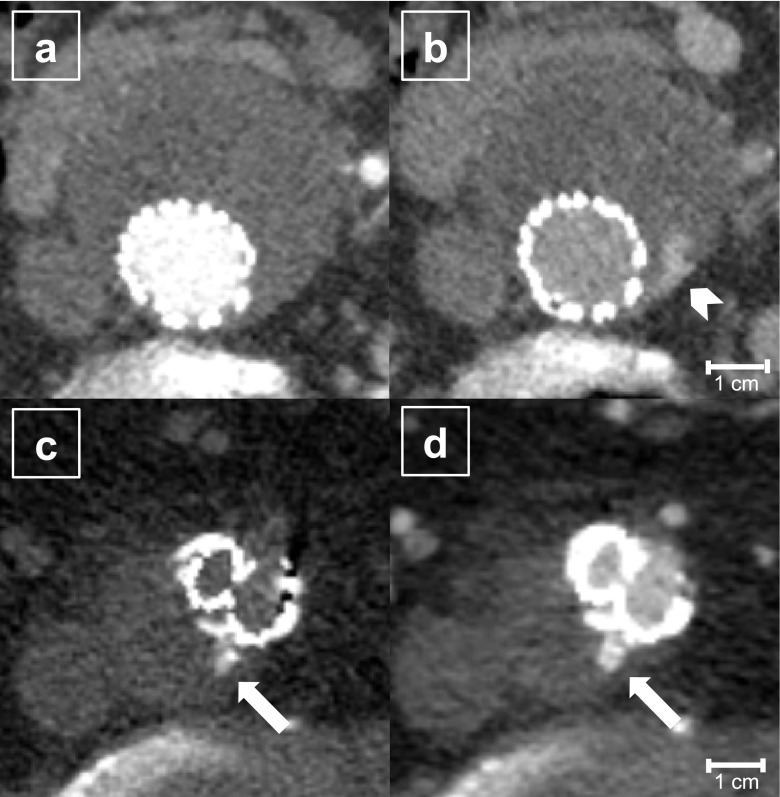
An 82-year-old EVAR patient (m) undergoing follow-up with a triphasic CT scan (level 100 HU, window 600 HU) 1 year after intervention. The arterial phase (**a**) and venous phase (**b**) show a low flow endoleak (arrowhead) apparent only in the venous phase (**b**). The native scan (**c**) identifies hyperdense material within the aneurysm sac as calcifications (arrows). These could be mistaken as an endoleak in the contrast-enhanced scan (**d**)

The Society for Vascular Surgery recommends life-long follow-ups (1, 6 and 12 months after the intervention and annually thereafter) using contrast-enhanced computed tomography (CT) scans to detect possible complications related to EVAR [9]. Because of different flow rates, endoleaks can manifest in the arterial (high flow) and/or in the venous or delayed phase (60–120 s post injection) (low flow) and might be difficult to distinguish from intra-aneurysmatic calcifications (Fig. 1). Therefore, standard CT protocols include a native scan of the abdomen (or just the stent graft area recognized by the survey scan), a scan of the whole abdomen in the arterial phase after intravenous contrast agent application and a delayed scan of the whole abdomen or stent graft area [10, 11]. A CT scan in the arterial phase also enables the evaluation of the access vessels and an in-stent lumen visualization, whereby organ infarction—a potential adverse effect of vascular intervention—is usually reliably detectable in the delayed phase scan.

However, repetitive multiphasic CT scans lead to a critical accumulative radiation dose. Dual-energy CT (DECT) allows decomposition into two basis materials and offers the possibility to reduce radiation burden in comparison to multiphase studies e.g. by providing a virtual non-contrast image [12]. The introduction of the current preclinical technique of spectral photon-counting CT (SPCCT) [13–17] could even further reduce radiation dose for patients under surveillance following EVAR. Spectral photon-counting detectors employed in SPCCT count incoming photons, bin them with respect to their energy and are therefore able to discriminate more than two different materials [18–20]. In addition, SPCCT is capable of ultra-high resolution imaging, improved signal-to-noise (SNR) and reduction of beam hardening artefacts.

**Fig. 2:**
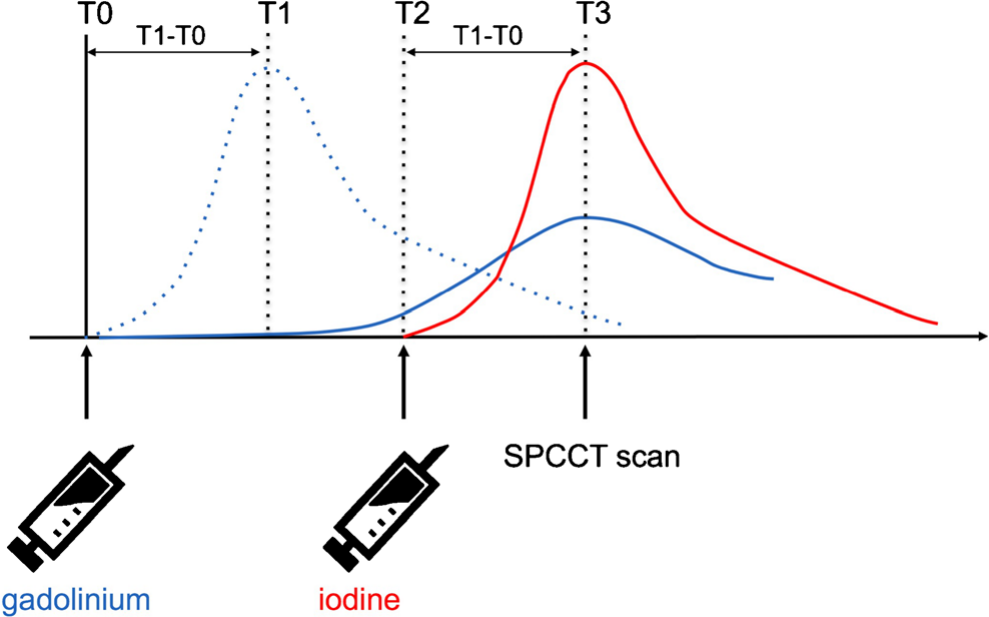
Dual contrast agent injection protocol for endoleak detection by a single SPCCT scan. Peak enhancement by gadolinium, injected at T0, should be registered (T1) within the lumen of the stent-graft. The time period T1–T0 defines the time necessary for maximal enhancement in the arterial phase. Under consideration of T1–T0, iodine should be injected (T2) in order to meet a venous/delayed distribution for the initially applied gadolinium and an arterial distribution of iodine, when a single SPCCT scan is performed at T3 = T2 + T1–T0. (Blue dotted line, arterial distribution of gadolinium; solid blue line, venous/delayed distribution of gadolinium; red line, arterial distribution of iodine)

Surveillance of EVAR patients with SPCCT will require a dedicated injection protocol for intravenous application of two contrast agents (e.g. gadolinium and iodine) (Fig. 2). A region of interest (ROI) for automated detection of contrast enhancement should be placed within the lumen of the stent-graft, and peak enhancement by gadolinium, injected at T0, should be registered (T1). Hence, the period T1–T0 defines the time necessary for a maximal enhancement in the arterial phase and it is an individual parameter that varies from patient to patient in relation to, among others, their cardiac output and aortic diameter. While the attenuation by gadolinium within the lumen of the stent-graft will decrease, the attenuation within the aneurysm sac will increase because of the endoleak. Under consideration of T1–T0 iodine should be injected (T2) in order to meet a venous/delayed phase (60–120 s post injection) for the initially applied gadolinium and an arterial distribution of iodine, when a single SPCCT scan is performed at T3 = T2 + T1–T0.

In this study, we demonstrate in a phantom model that a single SPCCT scan could capture endoleak dynamics and discriminate endoleaks from intra-aneurysmatic calcifications.

## Material and methods

### Aortic aneurysm phantom

An aortic aneurysm phantom was designed in-house and produced by a selective laser sintering of polyamide. The phantom was based on a conventional CT scan obtained in clinical routine following EVAR and planned in due consideration of our injection protocol (Fig. 2). The phantom contains eight compartments of different sizes and configurations arranged as tubes on a closed base. A centrally located compartment is lined up with a 36 mm × 100 mm covered stent-graft (Endurant, Medtronic, Germany) and filled with distilled water, iodine (Solutrast 370, Bracco, Germany; 370 mg iodine/ml) and gadolinium (Magnograf 0.5 mmol/ml, Jenapharm, Germany; 78.63 mg gadolinium/ml) mimicking enhanced blood within the stent lumen. Immediately adjacent to it, there is a compartment containing iodine (6 mg iodine/ml; Solutrast diluted in distilled water) that represents the leaking contrast media in the arterial phase injected at T2. Surrounding these, a gadolinium-filled compartment represents leaking in the venous/delayed phase (4 mg gadolinium/ml; Magnograf diluted in distilled water). Interposed calcium chloride (75 mg calcium chloride/ml; diluted in distilled water)-filled compartments mimic intra-aneurysmatic calcifications. Compartment 5 represents the excluded aneurysm sac and was filled with an oral contrast media used regularly in magnetic resonance imaging (Lumivision, Bender, Germany) to mimic clotted blood. Lumivision provides Hounsfield unit (HU) values ranging from 40 to 75 HU, which are frequently clinically measured within the excluded aneurysm sac following EVAR.

Likewise, the intensities of all components were based on HU measured in a routinely obtained aortic CT scan. Solutrast, Magnograf and calcium chloride were diluted in distilled water until similar intensities (approx. 150 HU at a tube voltage of 120 kVp) were reached. Solutions were shaken immediately prior to the SPCCT scan to avoid sedimentation.

### Spectral photon-counting CT examination

The phantom was scanned with a multi-bin preclinical SPCCT system (Philips Healthcare, Haifa, Israel) using a step-and-shoot acquisition mode with an X-ray tube current of 100 mA, an X-ray tube voltage of 120 kVp and a scanner rotation time of 1 s. The scanner is based on a clinical CT system (Brilliance iCT, Philips Healthcare, Haifa, Israel) providing a conventional X-ray tube and standard beam filtration but with a limited in-plane field of view of 168 mm and a z-coverage of 2.5 mm at isocentre. The hybrid photon-counting detectors ChromAIX2 ASICs (application-specific integrated circuit) [21] combined with cadmium zinc telluride (CZT) as sensor material have the capability to set five energy threshold values. The threshold positions were optimized to maximize the image signal-to-noise ratio [22]. They were set to 30, 51, 64, 72 and 85 keV in order to achieve high sensitivity for the different materials scanned, including gadolinium, which has a K-edge at 50.24 keV.

### Material decomposition, processing and image reconstruction

**Fig. 3:**
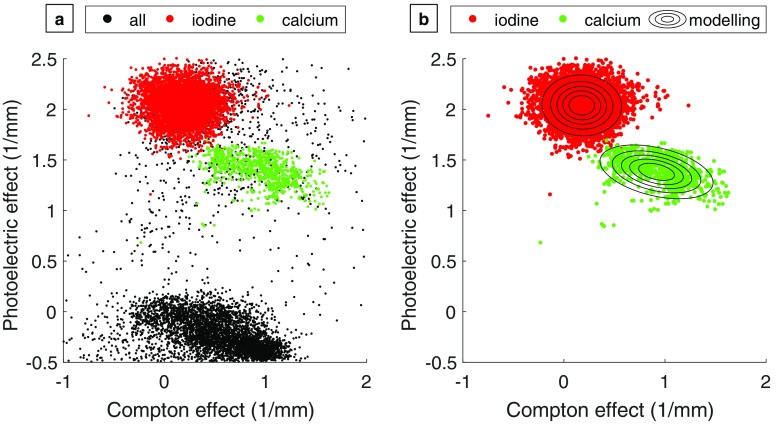
Image-based separation of calcium and iodine: distribution of photoelectric effect and Compton effect image (**a**) and modelling of material distributions of iodine and calcium (**b**)

A conventional image data set was reconstructed from pre-processed photon counts of all energy bins using filtered back-projection. The processing included a metal artefact reduction. Apart from this, two binary material maps were generated comprising the information whether an image voxel of the conventional image is more likely to contain iodine or calcium. To obtain the information about the material distributions within the phantom, the pre-processed photon counts were decomposed into three projection data sets of photoelectric effect, Compton effect and gadolinium by a maximum-likelihood approach [23]. The projections were separately reconstructed to images individually again by conventional filtered back-projection. For iodine and calcium, probability distributions were calculated from the images of the photoelectric effect and the Compton effect. The X-ray attenuation of different materials without K-edge absorption in the selected energy range can be described by a varying amount of attenuation by the photoelectric effect and the Compton effect [24] as illustrated in Fig. 3a. By evaluating for each image voxel the values in the photoelectric and Compton images, an image-based material separation of iodine and calcium was possible. The distributions of iodine and calcium both cluster around different mean value and were modelled by joint real normal distributions (Fig. 3b). By evaluating the distribution models for each image voxel probability maps for iodine and calcium were calculated. The images were statistically filtered taking into account the anti-correlated noise to reduce the overlap of the formed clusters [25]. For each image voxel, a binary decision was made whether the voxel contained calcium or iodine on the basis of the probability maps.

### Data analysis

The iodine and gadolinium concentrations were determined experimentally by material decomposition and by conversion of the CT values in the conventional image to concentrations via a look-up table. CT values of iodine and gadolinium and their concentrations obtained from the material-specific maps were measured by a single observer. Regions of interest (ROIs) where drawn with identical size and position in the conventional CT images as well as in the material maps. To demonstrate the visually improved differentiation between the different compartments a line profile representing the values in conventional and material maps was drawn. For improved comparability, the different images were scaled between zero and one for visualization of the line profiles.

## Results

**Fig. 4:**
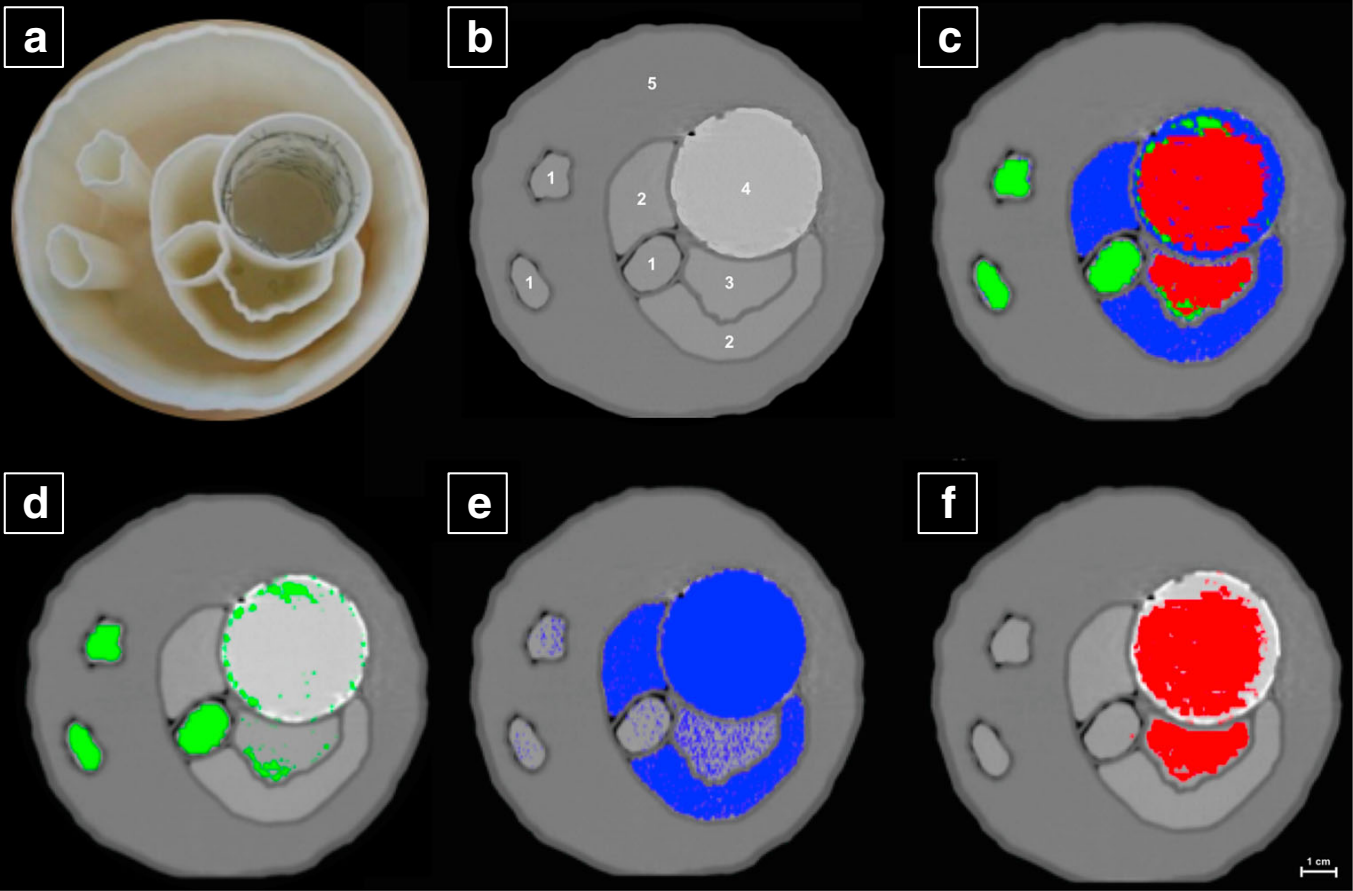
Photograph of the aortic phantom (**a**), conventional CT scan (**b**), overlay of the three material maps (**c**) (green = calcium; red = iodine; blue = gadolinium), calcium map (**d**), gadolinium map (**e**) and iodine map (**f**) (level −153 HU, window 1120 HU). 1 = calcium; 2 = gadolinium; 3 = iodine; 4 = stent lined, iodine and gadolinium. 5 = oral contrast media

Figure 4 shows a photographic image (a) as well as axial tomographic images (b–f) of the aortic phantom. Material-specific maps were created as an overlay of all material maps (c) and separately for calcium (d), gadolinium (e) and iodine (f). Measured in the conventional CT (Fig. 4b), the intensity of the ROIs placed in different compartments 1–5 were 151 HU, 147 HU, 168 HU, 340 HU and 40 HU with a standard deviation of 8 HU at 3 mm slice width, respectively. Of note, the iodine, gadolinium and calcium compartments were indistinguishable in Fig. 4b, illustrating a frequently occurring problem in clinical routine. On the SPCCT data, areas of the phantom containing gadolinium could be clearly identified on the basis of their characteristic increase of attenuation at the K-edge. Iodine and calcium probability maps—based on photoelectric effect and Compton effect—showed a slight overlap at given material concentrations and applied dose. However, both compartments were clearly assignable within the phantom.

The estimated concentration of iodine based on the CT value of 147 ± 3 HU was 4.9 ± 0.1 mg/ml and the estimated concentration of gadolinium based on 168 ± 4 HU was 3.5 ± 0.1 mg/ml. Using material decomposition, we measured the concentration of iodine as 3.6 ± 0.2 mg/ml and the concentration of gadolinium as 3.2 ± 0.1 mg/ml. Comparable errors of 1.2 mg/ml are also reported and discussed by Cormode et al. [17].

**Fig. 5:**
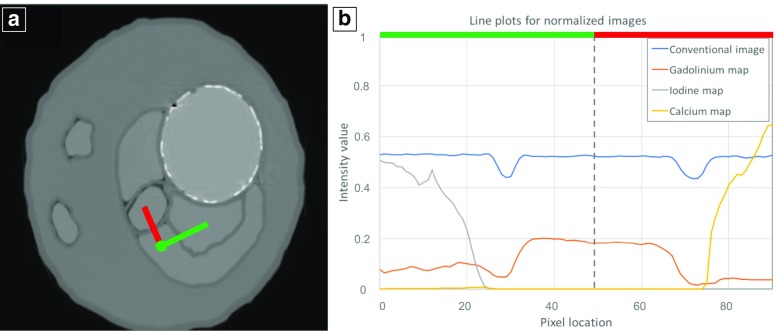
**a** Normalized conventional greyscale image of the aortic aneurysm phantom obtained with SPCCT. **b** Line plots, indicated in **a** with red and green lines, are tracking through the compartments filled with iodine, gadolinium and calcium. Intensities measured in the conventional SPCCT image (blue line), measured in the gadolinium map (orange line), obtained from the iodine probability map (grey line) and the calcium probability map (yellow line) are displayed

Figure 5 illustrates the improved differentiability when comparing conventional CT versus material-specific maps. The paths through the compartments filled with iodine, gadolinium and calcium are indicated with red and green lines in Fig. 5a. Figure 5b shows that with similar HU values in the conventional CT, the differentiation is impossible; whereas, it is possible with the material-specific maps. For improved comparability, the different images were scaled between zero and one for visualization of the line profiles.

## Discussion

This study provides first proof-of-concept results for SPCCT combined with a dedicated dual contrast agent application for reliable endoleak detection with a single CT acquisition, potentially lowering radiation dose for patients under surveillance following EVAR.

Since intra-aneurysmatic calcifications might be difficult to distinguish from leaking contrast media and endoleaks possess different flow rates, triphasic CT protocols are indispensable but add up to a significant radiation burden for the patient. Kalef-Ezra et al. point out an effective dose of 62 mSv only in the first year following EVAR [26]. In 2013 Brägelmann et al. reported a cumulative dose of 18.67 mSv for a triphasic CT protocol, consisting of a native (4.53 mSv), an arterial (7.13 mSv) and a delayed phase (7.01 mSv) scan [12]. Brägelmann et al. were able to show a dose reduction to 14.14 mSv by replacing the true native scan with a virtual native scan using a DECT scanner. Furthermore, the acquisition of only one single DECT scan in the delayed phase enabled an effective dose reduction of 62%, but can only provide limited information about the arterial flow path. Recently published work proposes a single-acquisition split-bolus DECT [27] providing the possibility to capture biphasic information within a single CT scan. Javor et al.’s analysis showed a significant reduction of radiation burden of up to 42%, while maintaining a sufficient diagnostic quality with an endoleak detection rate of 96% [27]. Unfortunately, the type of endoleak (either low or high flow) cannot be precisely determined, since with mixed arterio-venous contrast leakage is not assignable to the arterial or venous phase.

With the introduction of SPCCT, the possibility arises to answer clinically relevant questions by distinguishing two contrast agents. The important advantages of a SPCCT, compared to current CT technology, are based on the concept that incoming photons are counted with respect to their energy within predefined energy windows [18–20]. This technology offers the possibility to perform multi-material decomposition including K-edge imaging. Initial pilot studies have demonstrated the possibility to employ SPCCT data for diagnostic tasks [14–16, 21, 28–30]. Muenzel et al. demonstrated the feasibility of SPCCT-colonography to differentiate iodine-tagged faeces or fluids and gadolinium-enhanced polyps. Material maps clearly differentiated the distributions of gadolinium and iodine, and quantitative measurements of the material concentrations could also be performed with high accuracy [15]. Symons et al. successfully performed the decomposition of three contrast agents in a large animal model SPCCT scan. Distribution of intravenous successively administered gadolinium and iodine and oral applied bismuth, indistinguishable on the grayscale images, could be clearly differentiated with the SPCCT images. Symons et al. propose that, split-bolus dual-contrast SPCCT for multiphase kidney imaging could reduce radiation burden in comparison to conventional multiphase CT imaging [28].

Along the same lines, a single SPCCT scan in combination with a successive application of intravenous applied contrast media (e.g. iodine and gadolinium) can provide spatial and temporal information to detect endoleaks following EVAR. It allows observation of flow dynamics as well as the reliable distinction of leaking contrast media and intra-aneurysmatic calcifications, since identification of more than two materials is enabled. With respect to the diagnostic image quality, an improvement of spatial resolution, SNR and beam-hardening artefacts reduction can be expected. Additional misalignment of conventional CT scans in native, arterial and venous phase due to movement or breathing by the patient will be avoided. Consequently, by applying just one scan with an X-ray dose comparable to a single scan, SPCCT could significantly reduce the radiation burden for patients under surveillance following EVAR without sacrificing the diagnostic accuracy or may even improve it.

Presently, simultaneous applications of the two contrast agents iodine and gadolinium is not approved and needs pharmacological evaluation before the proposed injection protocol can be applied for patient care. Furthermore, current finding of depletions of specific (e.g. linear) gadolinium contrast agent in the dentate nucleus and pallidum after repetitive application, even in patients without renal impairment [31], questions the utilization of gadolinium-containing contrast media for novel clinical applications. However, the suggested injection protocol could be universally usable for various contrast media. Our injection protocol even provides the possibility to minimize the amount of applied contrast agent at the time point T2, because the triggering of the initially applied contrast agent allows reliable assessment of optimal enhancement and scan time. Therefore, dosage of contrast agent applied at T2 can be adjusted and potential side effects probably reduced.

The minimum of detectable leaking contrast media and minimal amount of discriminable contrast media towards intra-aneurysmatic calcifications cannot be assessed in the context of our experimental set-up. Calcium and iodine maps showed a slight overlap and this effect has to be further evaluated and revealed in vivo. Utilization of two K-edge materials would allow an easier and more reliable discrimination towards calcifications with high SNR.

With regard to the possibilities of SPCCT, K-edge imaging-compatible contrast agents would be of particular interest. Unfortunately, clinically approved iodine-based contrast agents have a relatively low K-edge (33.17 keV), which is unfeasible for K-edge imaging in clinical routine because of absorption of low energy photons below the K-edge. Therefore, the development and further promotion of new contrast agents, which are partially on the product roadmap [32], is of particular interest to fully exploit the benefits of SPCCT imaging.

## Conclusion

SPCCT in combination with a dual contrast agent injection protocol may replace conventional CT scans in native, arterial and delayed phase to detect endoleaks without sacrificing the diagnostic accuracy. It is a unique feature of our method to capture endoleak dynamics and allow distinction from intra-aneurysmatic calcifications in a single scan. This novel approach will potentially realize a relevant reduction of the radiation burden for patients under CT surveillance following EVAR.

## Abbreviations

AAA: Abdominal aortic aneurysm
ASIC: Application-specific integrated circuit
CT: Computed tomography
CZT: Cadmium zinc telluride
DECT: Dual-energy computed tomography
EVAR: Endovascular aortic repair
HU: Hounsfield units
MD: Material decomposition
SNR: Signal-to-noise ratio
SPCCT: Spectral photon-counting computed tomography
